# Temporal regularization of ultrasound‐based liver motion estimation for image‐guided radiation therapy

**DOI:** 10.1118/1.4938582

**Published:** 2016-11-30

**Authors:** Tuathan P. O'Shea, Jeffrey C. Bamber, Emma J. Harris

**Affiliations:** ^1^ Joint Department of Physics, The Institute of Cancer Research and The Royal Marsden NHS foundation Trust, Sutton, London SM2 5PT, United Kingdom

**Keywords:** biological tissues, biomedical ultrasonics, blood vessels, correlation methods, filtering theory, image matching, image sequences, linear accelerators, liver, medical image processing, motion estimation, radiation therapy, Ultrasonography, Treatment strategy, Diagnosis using ultrasonic, sonic or infrasonic waves, Radiation therapy, Biological material, e.g. blood, urine; Haemocytometers, Digital computing or data processing equipment or methods, specially adapted for specific applications, Image data processing or generation, in general, Analysis of motion, Linear accelerators, ultrasound, liver, motion, tracking, Ultrasonography, Liver, Motion estimation, Ultrasonic transducers, Image guided radiation therapy, Speckle, Transducer arrays, Sequence analysis, Image scanners

## Abstract

**Purpose:**

Ultrasound‐based motion estimation is an expanding subfield of image‐guided radiation therapy. Although ultrasound can detect tissue motion that is a fraction of a millimeter, its accuracy is variable. For controlling linear accelerator tracking and gating, ultrasound motion estimates must remain highly accurate throughout the imaging sequence. This study presents a temporal regularization method for correlation‐based template matching which aims to improve the accuracy of motion estimates.

**Methods:**

Liver ultrasound sequences (15–23 Hz imaging rate, 2.5–5.5 min length) from ten healthy volunteers under free breathing were used. Anatomical features (blood vessels) in each sequence were manually annotated for comparison with normalized cross‐correlation based template matching. Five sequences from a Siemens Acuson™ scanner were used for algorithm development (training set). Results from incremental tracking (IT) were compared with a temporal regularization method, which included a highly specific similarity metric and state observer, known as the *α*–*β* filter/similarity threshold (ABST). A further five sequences from an Elekta Clarity™ system were used for validation, without alteration of the tracking algorithm (validation set).

**Results:**

Overall, the ABST method produced marked improvements in vessel tracking accuracy. For the training set, the mean and 95th percentile (95%) errors (defined as the difference from manual annotations) were 1.6 and 1.4 mm, respectively (compared to 6.2 and 9.1 mm, respectively, for IT). For each sequence, the use of the state observer leads to improvement in the 95% error. For the validation set, the mean and 95% errors for the ABST method were 0.8 and 1.5 mm, respectively.

**Conclusions:**

Ultrasound‐based motion estimation has potential to monitor liver translation over long time periods with high accuracy. Nonrigid motion (strain) and the quality of the ultrasound data are likely to have an impact on tracking performance. A future study will investigate spatial uniformity of motion and its effect on the motion estimation errors.

## 1. INTRODUCTION

Internal tissue motion is known to compromise external beam radiation therapy (RT) delivery.^1^ Patient respiration is a particular challenge for treatment sites such as the lung and liver.^2^ Imaging‐based respiratory motion compensation strategies generally use kilo‐voltage x‐rays and often assume correlation between external surrogates and internal motion.^3^, ^4^ Ultrasound has been explored for use in both inter‐ and intra‐fraction motion compensation strategies and its use in RT has recently been reviewed.^5^ Benefits of ultrasound‐based image‐guided RT (IGRT) include (i) no ionizing radiation,^6^ (ii) no implantation of fiducial markers, and (iii) potential for high volumetric imaging rate (∼kHz for matrix transducer technology^7^). Ultrasound can also provide volumetric soft‐tissue data with no need for external surrogates.

In B‐mode images (without contrast agents), liver lesions often lack contrast relative to surrounding liver tissue^8^ and while tracking locally homogeneous echo texture is an option, Schlosser *et al.* ^9^ reported results which support the hypothesis that tracking an internal target, such as a liver blood vessel close to the tumor using ultrasound, is a more accurate surrogate than external infrared markers.

Correlation‐based (direct echo) estimation of liver motion in ultrasound data has been investigated for both locally homogeneous echo texture and clearly resolved liver features (blood vessels).^7^, ^10^, ^11^ In addition, good reproducibility of robotic probe placement for subcostal liver imaging has recently been reported.^12^ A 3D swept‐array transducer has been used to estimate liver feature motion *in vivo*, with good accuracy (mean absolute difference <2 mm).^10^ The complex nature of liver tissue motion (i.e., due to deformation and rotation)^13^ meant that naïve correlation‐based speckle tracking was not feasible at the low volume rates (0.5 Hz) of the mechanically swept probe. Bell *et al.* ^7^ used a 2D matrix array transducer to acquire liver motion data from three volunteers and track speckle in 3D at a volume rate of up to 48 Hz. Volumetric data were acquired without the imaging‐rate restrictions of a mechanical sweep. It was found that optimal volume rates of 8–12 Hz were required to accurately track cardiac and respiratory‐induced liver motion. A median filter‐based spatial regularization approach was employed to improve the mean accuracy of 3D displacement maps.

De Luca *et al.* ^11^ developed a scale‐adaptive block‐matching approach to feature tracking in long (200–600 s) 2D ultrasound sequences. The authors astutely note that while many correlation‐based methods have been proposed, their accuracy has only been tested on relatively short ultrasound sequences. Correlation‐based tracking might fail in long sequences due to inappropriate region‐of‐interest (ROI) size selection, changes in image similarity, and error accumulation. The reported method achieved an accuracy (mean absolute difference) of <1 mm for nine volunteer sequences, with improvements demonstrated over naïve correlation‐based tracking.

Motion estimation in long ultrasound sequences for controlling linear accelerator gating and tracking will require methods which provide high confidence in the output data stream. The system must continually monitor the quality of motion estimation data and notify the user if the quality of tracking results is low, for example, due to the target moving out of the field of view or changes in target appearance. In such cases, treatment must be interrupted and images assessed. Regularization can be used to solve ill‐posed problems or prevent overfitting by introducing additional information (e.g., penalty terms) for extreme parameter values (e.g., low image similarities, larger than expected interimage motion). Gastounioti *et al.* ^14^ demonstrated arterial wall motion estimation accuracy improvements when correlation‐based tracking was combined with a state observer (i.e., a Kalman filter).^15^ The current study investigates the use of temporally regularized liver feature (blood vessel) motion estimation in 2D ultrasound for image‐guided radiation therapy. Temporal regularization was achieved with the combination of (i) a similarity metric with high specificity and (ii) a fast and simplified form of the state observer for motion estimation, data smoothing (error handling), and control applications, known as the *α*–*β* filter.^16^, ^17^, ^18^ The temporally regularized correlation‐based tracking algorithm developed using a set of five training sequences was applied to a further five long *in vivo* 2D ultrasound validation sequences. Improvements in liver feature motion estimation accuracy were quantified by calculation of mean, maximum, and 95% errors.

## 2. MATERIALS AND METHODS

### 2.A. Ultrasound data

**Figure 1:**
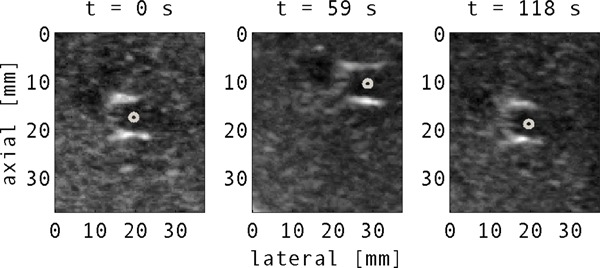
Typical vessel appearance within a fixed (100 × 100 pixel) search area at different times. The vessel center is manually annotated in each of the frames.

Ten 2D B‐mode ultrasound sequences acquired under free breathing were used. Data were acquired using an Acuson™ scanner (Antares; Siemens Medical Solutions, CA, USA) with convex curvilinear ultrasound transducer array (*f*_0_ of 1.82–2.22 MHz)^11^, ^19^ or a Clarity™ system (Elekta Ltd., Montreal, Canada) with an abdominal Autoscan™ 3D imaging transducer (*f*_0_ of 4.5 MHz) containing a convex curvilinear array operating in 2D mode. Sequences were acquired at imaging rates of 15–17 Hz (Acuson™) and 19–23 Hz (Clarity™) for 2.5–5.5 min. Mean ultrasound image pixel size was 0.44 mm over all sequences. Blood vessels (features) were identified in each sequence and manually annotated by an author (O'Shea) for comparison with the automated tracking code. The five sequences from the Acuson™ scanner were used for algorithm development (training dataset: se1–se5). A further five sequences (from the Clarity™ system) were used for validation, without alteration of the tracking algorithm parameters (validation dataset: se6–se10). For the training dataset, mean vessel cross‐sectional area and motion over the first 200 frames (3–4 breathing cycles) were analyzed, as presented in Table I. An example image showing the typical change in vessel appearance during various time intervals (for se5) is shown in Fig. 1.

**Table I:** Acquisition details for ten B‐mode ultrasound sequences (se1–se10) and analysis of feature motion in the training set (se1–se5) for the first 200 frames (∼2 breathing cycles).

Dataset	*f*_0_(MHz)	Pixel resolution (mm)	Frame rate (Hz)		Amplitude (mm)		Period (s)	Area (cm^2^)
				*X*/lateral	*Y*/axial	2D		
Training								
se1	2.00	0.40	16	8.4	2.1	8.6	3.4	10.7
se2	1.82	0.36	17	11.4	4.9	12.4	2.7	12.5
se3	2.22	0.42	15	40.1	5.1	40.4	5.5	16.7
se4	2.22	0.40	15	1.8	7.6	7.9	3.8	7.5
se5	1.82	0.37	17	8.3	7.6	11.2	4.7	21.2
			Mean	14.0	5.4	16.1	4.0	13.7
			Standard deviation	15.0	2.3	13.7	1.1	5.3
Validation								
se6	4.50	0.49	20					
se7	4.50	0.48	23					
se8	4.50	0.48	23					
se9	4.50	0.48	23					
se10	4.50	0.55	19					

### 2.B. Tracking code description

#### 2.B.1. Basic template matching and naïve incremental tracking (IT)

An IT program which used the location of the peak in a 2D (spatial) normalized cross‐correlation (NCC) function as a motion estimation metric was developed for the purpose of automated tracking of liver features. The program was written in matlab (7.13.0.564, Mathworks, Inc., Natick, MA, USA). Subpixel displacement estimates were obtained by fitting a 1D second order function to the peak of the correlation matrix and two surrounding values, along both the axial and lateral directions. The peak of the parabolic fit (i.e., when slope, *m* = 0) was then used to indicate the subpixel displacement.

A point‐of‐interest (POI), ROI centered around the POI (in frame no. 1), and search region (SR) (for subsequent frames) were initially selected by the user. The selected POI was the center of the blood vessel to be tracked. The ROI size (22 × 22 mm^2^) was set to fully enclose each tracked vessel within all sequences. For (cumulative interframe) IT, the POI was dynamically updated in each frame according to the position estimate from the previous frame. The SR was a larger region selected in a subsequent frame in which a normalized correlation search was performed to locate the ROI defined in the current frame. The SR was set to encompass a maximum range of interframe motion (44 × 44 mm^2^) much larger than expected liver motion. For incremental tracking, ROI motion was tracked between frame nos. 1 and 2, 2 and 3, etc. For fixed ROI (nonincremental) tracking, the interframe image displacement will be larger and the displacement estimates are expected to be less precise; however, accuracy can be maintained over longer sequences provided the tissue does not translate, rotate, or deform substantially. For incremental tracking, error accumulation has been identified as a significant drawback,^10^ but the method has potential for tracking features which change appearance substantially in longer sequences.

#### 2.B.2. Similarity metric and point‐of‐interest update

The motion estimation output from the above naive template‐matching algorithm may be inaccurate due to factors such as electronic noise, out‐of‐plane motion, tissue deformation, tissue rotation, and subsample bias. Using this output directly to update the POI can produce tracking errors. This leads to a particular challenge in vessel tracking: how to deal with the gradual change in target appearance (over the entire sequence or indeed the breathing cycle) that reduces interimage correlation. To maintain accurate vessel tracking, the POI may need to be updated more robustly (accurately) than as described above. Using naïve incremental tracking methods (i.e., where the POI is updated every frame), bias in subpixel displacement estimates can accumulate over many frames and lead to substantial underestimation of vessel motion amplitude (see Ref. 11). In the current study, a POI update strategy was investigated whereby the POI was updated only when an image similarity metric dropped below a user‐defined threshold, referred to as the similarity threshold (ST). At this point, it was determined that the feature appearance had change substantially and the POI was updated.

**Figure 2:**
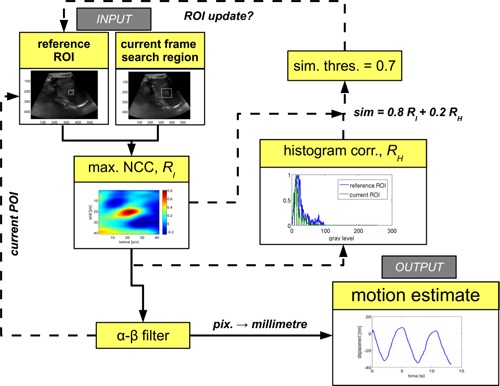
Overview of the motion estimation algorithm. Naive incremental tracking (Sec. 2.B.1) used 2D NCC to track POI motion, with the reference ROI updated every frame. The peak of the 2D NCC was used to determine the new POI position. For similarity threshold based ROI updating (Sec. 2.B.2), the normalized correlation of the reference ROI and current ROI (centered on the new POI) histogram (*R_H_*) was combined with the NCC peak [*R_I_*, using Eq. (1)] and used to determine whether to update the ROI (via a threshold value). Finally, the displacement estimate (POI position) determined by the NCC peak was filtered using the *α*–*β* prediction‐based filter which was used to determine both the center of the reference ROI and the current motion estimate (Sec. 2.B.3).

An ideal similarity metric is exclusively large only when two images are very similar. Using the NCC peak to indicate the target's new position may become inaccurate. For example, when objects of similar structure are close to the target vessel and the maximum correlation value corresponds to the similar but incorrectly tracked vessel (i.e., *a false‐match*). In order to enhance tracking robustness, a hybrid metric was therefore developed to determine the ROI and current target similarity. This similarity metric combined both structural similarity (spatial correlation) and gray‐scale similarity (histogram correlation),

(1) $$\text{similarity metric}\;(\text{sim})=\gamma R_{I}+\left(1-\gamma \right)R_{H},$$

where *R_I_* denotes the value at the peak of the spatial NCC function (NCC peak) and *R_H_* is gray‐scale similarity. Calculating the gray‐scale similarity, *R_H_*, involved computing the normalized correlation between the gray‐scale distributions of the current target ROI (centered on the POI position determined by the spatial NCC peak) and the previous template ROI. In Eq. (1), *γ* was a weighting factor in the combination of spatial and gray‐scale similarities. Analysis of fixed ROI (i.e., nonincremental) tracking for se4 showed that a *γ* value of ≥0.5 gave a tracking error sensitivity and specificity of 100% and 99.7%, respectively. A *γ* value of 1.0 (i.e., using spatial correlation only) resulted in a lower specificity of 93.9%. The integration of the similarity metric as a threshold for updating the ROI is illustrated in Fig. 2.

#### 2.B.3. Temporal regularization using modified *α*–*β* filter

The ST‐based POI update (Sec. 2.B.2) was combined with an *α*–*β* filter to implement a form of temporal regularization of the motion estimation. A derivative of the Kalman filter,^15^ the *α*–*β* filter is a fixed‐gain implementation which can be used to estimate the smoothed position and velocity of a system. The main objective of the filter is to reduce measurement noise when tracking a moving target. Useful properties of the *α*–*β* filter are that it does not require a detailed system model and can be implemented with low computational overhead. The filter is a one‐step ahead predictor which uses the error (between the current measurement and prediction) to provide an estimate of the current state (i.e., position, *x* and velocity, *v*). The prediction error is weighted by smoothing (gain) parameters *α* and *β*. These parameters influence the algorithm's ability to filter out (reject) noise but also estimate the position and velocity of a moving target. The optimal filter should be able to track a target in both transient and steady‐state conditions and tune itself to the moving object's motion characteristics. However, these are competing requirements and compromise settings are usually required. For example, a small value of *α* produces more noise reduction but decreases response to transients such as motion between peak inhale and peak exhale. In general, large *α* and *β* values produce faster response for tracking transient changes. Appropriate selection of gain parameters is therefore important.

The *α*–*β* filter assumes two system states (position *x* and velocity *v*). Assuming that velocity remains constant over the small time period *T* between measurements (which is a valid assumption at the high frame rates of the US sequences), the position state is updated to predict its value in the next frame (*n* + 1),

(2) $$x_{p}\left(n+1\right)=x_{s}\left(n\right)+v_{s}\left(n\right)T,$$

(3) $$v_{p}\left(n+1\right)=v_{s}\left(n\right),$$

where *x_p_*(*n* + 1) and *v_p_*(*n* + 1) are the predicted position and velocity in the next frame and *x_s_*(*n*) and *v_s_*(*n*) are the smoothed position and velocity in the current frame. The measured displacement will likely deviate from prediction as described in Sec. 2.B.2. The prediction error is defined as

(4) $$r\left(n\right)=x_{m}\left(n\right)-x_{p}\left(n\right),$$

where *x_m_*(*n*) is the NCC‐based position estimate and *x_p_*(*n*) is the predicted position. The smoothed estimates for the current position and velocity are given by

(5) $$x_{s}\left(n\right)=x_{p}\left(n\right)+\alpha \;r\left(n\right),$$

(6) $$v_{s}\left(n\right)=v_{p}\left(n\right)+\left(\beta /T\right)r\left(n\right).$$

The *α*–*β* filter uses *α* multiplied by the prediction error *r* to correct (or smooth) the position estimation. Likewise *β* is multiplied by *r* to correct the velocity estimate. Benedict and Bordner^20^ derived a relationship between *α* and *β* which optimized the filter's performance for tracking noisy data changing at a constant velocity,

(7) $$\beta =\alpha^{2}/\left(2-\alpha \right).$$

The *α*–*β* filter was implemented in the template‐matching code using two different techniques which were compared experimentally. First, the optimal *α* value was determined empirically for the five ultrasound sequences (by varying the value from 0.2 to 0.8 and calculating the mean and 95% errors compared to ground truth) and a constant *α* value was then used for all sequences. In a second implementation, the *α* value was allowed to varying as a function of time by performing a linear mapping of similarity (sim) to *α* value (limited to the range 0.2–0.8). As *α* → 0, more weight was placed on the predicted position, conversely as *α* → 1, more weight was placed on the measured position. Since similarity provides a measure of confidence in the tracking code output, a high sim value was mapped to a high *α* value, while a low sim value mapped to low *α*.

#### 2.B.4. Position prediction

In the *α*–*β* filter, the predicted position, *x_p_*(*n*), was assumed to follow linear kinematics:

(8) $$x_{p}\left(n\right)=x_{s}\left(n-1\right)+v_{s}\left(n-1\right)T.$$

This linear motion assumption was expected to be justified for the short imaging interval (60–70 ms) of the ultrasound sequences where interframe displacement was small and approximately linear. For comparison, the position prediction was also calculated by linear prediction, a method used extensively to account for RT system latencies (e.g., Ref. 21). Linear prediction uses a defined signal history length (SHL) to predict a future output signal as a linear function of known inputs,

(9) $$x_{p}\left(n\right)=a_{0}+a_{1}x_{s}\left(n-1\right)+\cdots +a_{i}x_{s}\left(n-i\right),$$

with *i* previous positions from *x_s_*(*n* − 1) to *x_s_*(*n* − *i*). For a specified SHL(*i*), optimal predictor coefficients *a*_0_–*a_i_* can be found using least squares methods to minimize the prediction error.^22^

**Figure 3:**
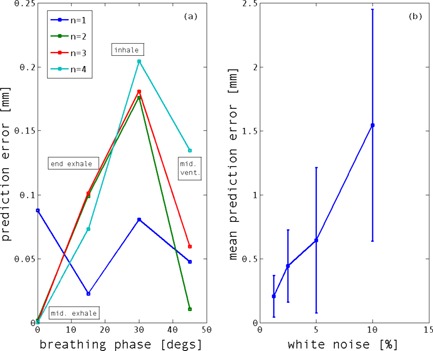
(a) Linear motion prediction error (for 70 ms latency) as a function of breathing phase for a simulated 15 Hz breathing signal with 16.1 mm amplitude and 4 s period [*n* is the flatness/steepness parameter in Eq. (10)]. (b) Mean (±S.D. for three simulations) prediction error (over all breathing phases for *n* = 2) as a function of maximum white noise level: ±1.25%, ±2.5%, ±5.0%, or ±10.0% of breathing signal amplitude.

Using simulated breathing motion signals derived from the equation presented by Lujan *et al.*,^23^

(10) $$x\left(t\right)=x_{0}-b\;\cos^{2n}\left(\pi t/\tau -\Phi \right),$$

it was also found that, while the accuracy [i.e., agreement between *x_p_*(*n*) and *x*(*t*)] of the linear motion assumption [Eq. (8)] was dependent on breathing phase, the maximum prediction error did not exceed 0.2 mm (at peak inhale for *n* = 1–4) for a signal amplitude of 16.1 mm and period of 4 s at 70 ms latency [Fig. 3(a)]. In Eq. (10), *x*_0_ is the position at exhale, *b* is the amplitude of motion, *τ* is the period of breathing, Φ is the starting phase, and *n* is the steepness/flatness shape parameter. The effect of noise on the prediction error was also simulated by adding white noise to the signal [Fig. 3(b)]. The use of the *α*–*β* filter to regularize the displacement estimates by combining measured and predicted displacements had the effect of reducing noise to less than 2% of the breathing amplitude.

### 2.C. Data analysis

The performance of the automated tracking method was evaluated by calculation of the mean absolute (±standard deviation), maximum and 95th percentile (95%) difference between manually annotated and tracking code motion estimates. An accuracy threshold of 2.0 mm for mean absolute and 95% difference was used, above which tracking was judged to have failed. For the training dataset, naïve IT was compared with the similarity threshold‐based POI update (ST) and a combined application of the similarity threshold and *α*–*β* filter/similarity threshold (ABST) methods. The accuracy of the final ABST method was then assessed for the validation dataset.

## 3. RESULTS

**Figure 4:**
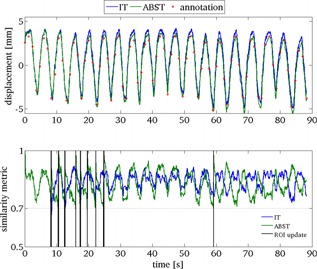
An example assessment of (i) naïve IT (grey line) and (ii) combined similarity threshold and alpha‐beta filter regularized motion estimation (ABST, light grey line) by comparison with manual annotations (points). The interframe similarity metric value is shown below. For IT, the POI was updated every frame. Conversely, for ABST, the POI position was updated using the state estimate, only when the similarity was below a threshold value (as indicated by the vertical black lines). The data presented are for a 90 s segment of vessel tracking from sequence se1.

Figure 4 shows a comparison of the naïve IT method and the combined ABST regularized tracking method, for a segment of the se1 training sequence (in the figure inhale has negative sign). It can be seen that the ABST method is in better agreement with manual annotations than the IT method. For IT, continual template (POI) update leads to error accumulation. The similarity value for each frame is also shown in Fig. 4. For ABST tracking, the POI was updated using the *α*–*β* filter displacement estimate only when the similarity value dropped below a threshold (0.7). This threshold value was empirically optimized for each of the five sequences. Time‐points of POI update are indicated by the black vertical lines in the lower graph of Fig. 4.

**Figure 5:**
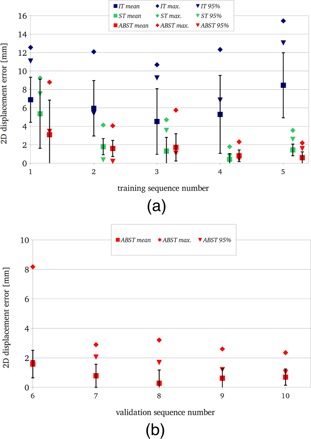
Accuracy (±standard deviation) of vessel displacement estimation in the five training ultrasound imaging sequences (se1–se5) for the three motion estimation methods: (i) IT—in which the POI was updated every frame, (ii) ST—where the POI was updated only when the similarity metric was below a threshold, and (iii) ABST—which combined the ST method with a state estimate predicted by the *α*–*β* filter (a). Accuracy (±standard deviation) of vessel displacement estimation for the five validation datasets (se6–se10) and the ABST method is shown below (b).

The overall accuracy (mean, standard deviation, maximum, and 95% errors compared to annotations) of the IT, ST, and ABST methods for the training set is compared in Table II and Fig. 5. IT failed to track the vessel motion in all sequences. The overall mean and 95% errors (6.2 and 9.1 mm, respectively) were much greater than the predefined successful tracking threshold (2.0 mm). Using the similarity metric which combined spatial and gray‐scale similarity for error detection and POI update produced large improvements in accuracy (mean: 2.1 mm, 95%: 3.0 mm). The ABST method (with fixed *α* value of 0.5) further improved overall vessel tracking accuracy (mean: 1.6 mm, 95%: 1.4 mm). To verify the linear motion prediction assumption, the *α*–*β* filter prediction stage was also implemented using linear prediction with a SHL = 4 s. This did not improve accuracy as shown in Table II. To enable automated *α*–*β* filter parameter selection, the use of the interframe similarity value to set filter gain was also investigated (as described in Sec. 2.B.3). The range (minimum to maximum) of interframe similarity values were linearly mapped to *α* values in the range of 0.2 (less confidence in measurement) to 0.8 (more confidence in measurement). As shown in Table II, this method did not improve accuracy compared to the optimized fixed gain implementation. Finally, to determine the effect of the subsample displacement estimation on accuracy, the vessel displacements were calculated to the nearest integer pixel displacement. This increased the overall 95% error by 1.3 mm.

**Table II:** Overall accuracy of various liver feature motion estimation methods for all training sequences: (i) IT—in which the POI was updated every frame, (ii) ST—where the POI was updated only when the similarity metric was below a threshold value, and (iii) ABST—which combined the similarity threshold with a state estimate predicted by the *α*–*β* filter. Using linear prediction [Eq. (9)] was not found to improve the performance of the ABST method [change in 95% error (Δ95%) compared to no modification: +0.3 mm]. The code was also run by linearly mapping the interframe similarity value to filter *α* value which also failed to improve accuracy. The integer displacement was calculated, without subsample displacement estimation, to determine the effect on accuracy (Δ95%: +1.3 mm). The accuracy of the ABST implementation for the validation dataset is included. Key data for each sequence are also plotted in Figs. 5(a) and 5(b).

			Tracking parameters^a^		2D displacement accuracy (mm)				
Set	Tracking method	Method modification	Similarity threshold	*α*	Mean	SD	Maximum	95%	Δ95%
Training	IT	—	—	—	6.2	3.4	15.4	9.1	—
	ST	—	0.7	—	2.1	1.1	9.2	3.0	−6.2
	ABST	None	0.7	0.5	1.6	1.5	8.8	1.4	−7.8
		Linear prediction^b^	0.7	0.5	1.9	1.8	8.7	1.7	0.3
		Sim → alpha	0.7	0.2–0.8	1.9	1.3	10.3	2.5	1.1
		Pixel interframe displacement	0.7	0.5	1.7	1.1	9.8	2.6	1.3
									
Validation	ABST	None	0.7	0.5	0.8	0.8	8.2	1.5	—

^a^ ROI = 50 × 50, srch = 100 × 100.

^b^ SHL = 4 s.

The accuracy of the ABST method for the validation data is also presented in Table II. The algorithm parameters were unadjusted from those used for the training set. The overall mean and 95% errors were 0.8 and 1.5 mm, respectively. The mean and 95% errors for each sequence ranged from 0.3 to 1.6 mm and 1.0 to 2.1 mm, respectively [Fig. 5(b)].

In Fig. 5(a), it can be seen that for four of the five training sequences (se2–se5), the tracking accuracy goal (mean/95% errors ≤2.0 mm) was achieved. The ST and ABST tracking methods improve the 95% errors (to <4 and <2 mm, respectively) for all five training sequences. For se1, the mean and 95% errors were 3.1 and 3.4 mm, respectively. For the validation dataset, the accuracy goal was also achieved for four of the five sequences. For se7, the 95% error was above the accuracy threshold.

For the liver, tissue deformation during the breathing cycle can be significant.^13^ This led to the hypothesis that the loss of correlation and thus similarity may be affected more by deformation for some training sequences (refer to the mean and minimum similarity values in Table III). The higher rate of decorrelation for se1 meant that the similarity threshold produced a high rate of POI update but potentially updated at an incorrect position (i.e., not the vessel center). Reducing either the similarity threshold (ST = 0.6, mean error: 1.2 mm; 95% error: 2.4 mm) or the alpha value (*α* = 0.2, mean error: 0.5 mm, 95% error: 6.7 mm) was found to improve tracking accuracy for se1.

**Figure 6:**
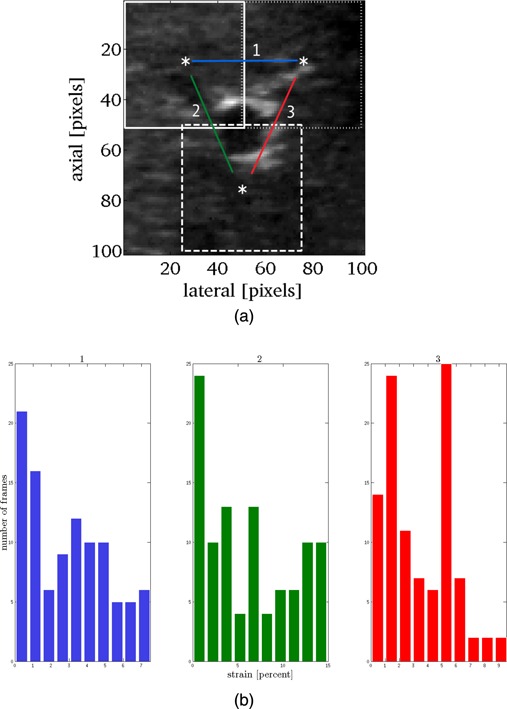
Tissue motion for each training sequence and the first 150 frames was calculated in three ROIs (white squares) as illustrated (a). The percent inter‐POI strain, ε (i.e., change in distance/initial distance: Δ*L*/*L*_0_) as a function of time (frame) was calculated and the mean 2D strain (%) was evaluated as an indicator of nonrigid motion (b).

**Table III:** Correlation of ABST mean and 95% tracking errors for each sequence with the parameters listed in row 1: mean and minimum similarity, vessel 2D motion amplitude and period, and vessel circular cross‐sectional area over the first 150 frames. The mean strain for 2D motion of the three ROIs illustrated in Fig. 6 for each ultrasound sequence is also included. A higher value indicates potential increased tissue deformation (and/or rotation). From the table, it is evident that the mean similarity is highly correlated with the mean (tracking) error (0.913) and the strain (0.846). Boldface values indicate high correlation.

Sequence	Mean similarity	Min. similarity	2D amplitude (mm)	Period (s)	Area (cm^2^)	Strain (%)
se1	0.742	0.470	8.6	3.4	10.7	15.1
se2	0.801	0.688	12.4	2.7	12.5	12.9
se3	0.764	0.456	40.4	5.5	16.7	12.0
se4	0.805	0.654	7.9	3.8	7.5	11.3
se5	0.837	0.666	11.2	4.7	21.2	6.3
Pearson correlation						
Mean error (mm)	**0.913**	**0.722**	0.060	0.277	0.319	**0.846**
95% error (mm)	0.563	0.628	0.161	0.042	0.064	0.285

To investigate potential vessel deformation, three ROIs positioned around the target vessel in each training sequence were tracked over the first 150 frames as indicated in Fig. 6(a). The mean absolute percentage strain (ε) in the inter‐POI distance was calculated as an indication of rigid body error or target deformation,

(11) $$\varepsilon =\left(\Delta L/L_{0}\right)\times 100,$$

where Δ*L* was the change in distance relative to the distance in frame 1 (*L*_0_). For the vessel in each sequence, the percentage strain between each POI and each of the other POI was calculated [i.e., POI no. 1 and POI no. 2 (1), POI no. 1 and POI no. 3 (2), and POI no. 2 and POI no. 3 (3)]. A low value was indicative of motion uniformity. A higher value was potentially due to increased tissue deformation.

Figure 6(b) illustrates the variation in strain for se5 (assuming accurate tracking over the 150 frames). For the ABST method, the mean error showed high Pearson correlation of 0.85, (Table III) with calculated strain value.

## 4. DISCUSSION

Previous studies have explored both fixed ROI and incremental tracking of homogeneous liver tissue (speckle) and clearly resolved liver features (blood vessels).^7^, ^10^ While fixed region‐of‐interest (template) NCC‐based motion estimation is an option for ultrasound‐based IGRT, the algorithm can only maintain accuracy over longer sequences provided the tissue does not change substantially (e.g., due to rotation, deformation, and/or out‐of‐plane motion). Conversely, for incremental tracking, error accumulation has been identified as a significant drawback to 3D tracking at low volume rates and fixed ROI tracking gave more accurate results (Harris *et al.*). Using a matrix array transducer operating at high volume rates (24 and 48 Hz), Bell *et al.* ^7^ incrementally tracked soft tissue (speckle) in 3D without temporal regularization using a reportedly unbiased subsample displacement estimate.^24^

Outside the medical ultrasound tissue motion‐estimation field, template update drift is a known issue (e.g., Ref. 25). For incremental tracking at the frame rates of the current study, the primary source of error will often be for displacements which are much less than the pixel length. In our data, naïve IT resulted in many underestimated displacements potentially due to bias errors (in the interpolated NCC peak position). Nonrigid motion, rotation, out‐of‐plane translation and the resultant decorrelation also likely have an impact when updating the POI. The similarity‐measure based POI update strategy, combined with a state estimate (ABST), was generally successful in mitigating these effects. Subsample displacement estimation was also found to be an important step for template update at the imaging rates investigated (15–23 Hz) as illustrated in Table II: without subsample displacement estimation, the motion‐estimation accuracy decreased.

This study used envelope‐detected and scan‐converted images with coarse spatial resolution (generally too large) relative to the small interframe displacements at the high sampling (image) rate (15–17 Hz) of respiratory and cardiac‐induced liver motion. In this case, the phase information in RF data may provide sensitivity to smaller displacements (and help reduce bias). It is hypothesized that for IGRT applications, the tracking accuracy improvement of RF data over the envelope‐detected signal is a function of imaging rate. At high imaging rates, the interframe displacements may be small enough for the use of RF data to improve accuracy but at lower imaging rates the reverse may be true, i.e., the envelope‐detected data may provide signal features which can be tracked at greater levels of translation, rotation, and deformation than can be tolerated by RF data. This would be consistent with the previous finding by Doyley *et al.* ^26^ who showed in strain imaging that RF tracking performed best for small strains and envelope tracking best for large strains. Varghese and Ophir^27^ also proposed a strain imaging algorithm that combined the use of RF signals for small strains and envelope signals for estimation of larger tissue strains. A similar approach may further improve tracking accuracy for liver vessel motion measurement.

Our tracking accuracy goal (of mean and 95% errors ≤2 mm) was achieved for four of the five individual training and validation sequences. For training sequence 1 (se1), the tracking accuracy was notably poorer than this. Nonrigid motion (deformation and/or rotation) was identified as a potential cause of the lower accuracy. As discussed by Harris *et al.*,^10^ it is expected that tracking accuracy will vary for different segments of the liver due to deformation^13^ and this likely also applies to the different vessel (sizes and locations). Different vessels may also be more prone to deformation.^28^ Se1 had the second smallest vessel cross‐sectional area but overall tracking error did not correlate with vessel c.s.a (Table III). Calculation of the strain for three ROIs placed around the vessel, as an indicator of vessel deformation, showed high correlation with mean tracking errors (Fig. 6 and Table III) indicating that for certain vessels (e.g., in sequence 1 or 6), deformation may compromise the motion estimation accuracy of the presented method. This points toward the need for a combined spatiotemporal regularization technique to extend the automated ABST tracking method to vessels or tissues experiencing nonrigid motion (e.g., larger deformation). One method could involve extending previous work in this area, where a displacement map comprising multiple ROI was calculated and used to implement a spatial regularization scheme (in that instance: median filtering).^7^

For development purposes, we attempted to optimize the tracking algorithms' parameters for all training ultrasound sequences, such that the overall tracking errors were the lowest. An alternative approach could use a training sequence containing a number of breathing cycles to either automatically determine tracking algorithm parameters or, with adequate training, a manually supervised training period prior to a test tracking session where the operator is prompted to verify or click on the vessel position at various time‐points. This may only require the individual to identify the vessel at four or five position over a 10 s period. For example, our therapists continually monitor the CyberKnife™ synchrony system (internal fiducial/external surrogate) correlation error and rebuild the model if required. The therapist can manually select points in time/phase on an indicative diagram of a “breathing” trace, to rebuild the model. Our analysis has also shown that we can achieve high precision between annotations by three (nontherapist) observers over the initial 25 frames or two breathing cycles for five sequences [2D mean absolute difference (±SD): 1.0 ± 0.7 mm, with agreement (mean ± SD) between two of the observers of 0.5 ± 0.4 mm].

Finally, 2D tracking will only enable accurate monitoring of vessel displacements provided that the vessel long‐axis is approximately perpendicular to the image plane and out‐of‐plane motion is small. A matrix array transducer operating at high volume rates would be the optimal solution to overcome these restrictions (by enabling fast 3D imaging) but currently this technology is underdeveloped.^7^ An alternative approach could use a model which relates a single pretreatment (or updated) 3D ultrasound volume or MR/CT data to the 2D imaging plane^29^ taking advantage of high frame rate 2D imaging during treatment. A pretreatment imaging stage could also be used to generate a correlation model between the tumor position (in a contrast‐enhanced B‐mode image and/or MR or CT image) and trackable features (e.g., blood vessels surrounding the tumor) to enable accurate internal surrogate tracking.

## 5. CONCLUSIONS

The use of template‐matching based motion estimation in liver ultrasound was investigated and an accurate automated vessel tracking method was developed. This largely overcame drift and error accumulation which otherwise caused vessel motion estimates to become inaccurate in incrementally tracked long imaging sequences which meant that vessel motion estimates became inaccurate were largely overcome. A high‐specificity error detection and region‐of‐interest (template) update metric was introduced to threshold the template update rate. This was combined with a state estimator that produced a combined similarity and prediction‐based motion estimate of high accuracy (overall mean and 95% tracking errors were 1.6 and 1.4 mm, respectively, for five training datasets). For four of the five training datasets, an accuracy goal of mean and 95% tracking errors ≤2 mm were achieved for sequences of up to 5 min 30 s in length. For the validation dataset, the overall mean and 95% errors were 0.8 and 1.5 mm. Similarly, the accuracy goal was achieved for four of the five datasets. The input image quality appears to be an important factor, as well as nonrigid tissue motion. A future study will investigate spatial uniformity of motion and its effect on the motion estimation errors in detail.

## ACKNOWLEDGMENTS

This work was supported by Cancer Research UK Grant No. C33589/A19727. The authors acknowledge NHS funding to the NIHR Biomedical Research Center at The Royal Marsden and The Institute of Cancer Research.

## References

[1] S. S. Korreman , “Motion in radiotherapy: Photon therapy,” Phys. Med. Biol. 57(23), R161–R191 (2012).10.1088/0031‐9155/57/23/R161 23165229 10.1088/0031-9155/57/23/R161

[2] P. J. Keall , G. S. Mageras , J. M. Balter , R. S. Emery , K. M. Forster , S. B. Jiang , and J. M. Kapatoes , “The management of respiratory motion in radiation oncology report of AAPM Task Group 76,” Med. Phys. 33(10), 3874–3900 (2006).10.1118/1.2349696 17089851 10.1118/1.2349696

[3] M. Hoogeman , J. B. Prévost , J. Nuyttens , J. Pöll , P. Levendag , and B. Heijmen , “Clinical accuracy of the respiratory tumor tracking system of the cyberknife: Assessment by analysis of log files,” Int. J. Radiat. Oncol., Biol., Phys. 74(1), 297–303 (2009).10.1016/j.ijrobp.2008.12.041 19362249 10.1016/j.ijrobp.2008.12.041

[4] Y. Seppenwoolde , R. I. Berbeco , S. Nishioka , H. Shirato , and B. Heijmen , “Accuracy of tumor motion compensation algorithm from a robotic respiratory tracking system: A simulation study,” Med. Phys. 34(7), 2774–2784 (2007).10.1118/1.2739811 17821984 10.1118/1.2739811

[5] D. Fontanarosa , S. van der Meer , J. Bamber , E. Harris , T. O'Shea , and F. Verhaegen , “Review of ultrasound image guidance in external beam radiotherapy: I. Treatment planning and inter‐fraction motion management,” Phys. Med. Biol. 60(3), R77–R114 (2015).10.1088/0031‐9155/60/3/R77 25592664 10.1088/0031-9155/60/3/R77

[6] M. J. Murphy , J. Balter , S. Balter , J. A. BenComo, Jr. , I. J. Das , S. B. Jiang , and F. F. Yin , “The management of imaging dose during image‐guided radiotherapy: Report of the AAPM Task Group 75,” Med. Phys. 34(10), 4041–4063 (2007).10.1118/1.2775667 17985650 10.1118/1.2775667

[7] M. A. L. Bell , B. C. Byram , E. J. Harris , P. M. Evans , and J. C. Bamber , “ *In vivo* liver tracking with a high volume rate 4D ultrasound scanner and a 2D matrix array probe,” Phys. Med. Biol. 57(5), 1359–1374 (2012).10.1088/0031‐9155/57/5/1359 22349408 10.1088/0031-9155/57/5/1359

[8] C. F. Dietrich , A. Ignee , J. Trojan , C. Fellbaum , and G. Schuessler , “Improved characterisation of histologically proven liver tumours by contrast enhanced ultrasonography during the portal venous and specific late phase of SHU 508A,” Gut 53(3), 401–405 (2004).10.1136/gut.2003.026260 14960524 10.1136/gut.2003.026260 PMC1773968

[9] J. Schlosser , K. Salisbury , and D. Hristov , “Image‐based approach to respiratory gating for liver radiotherapy using a telerobotic ultrasound system,” Int. J. Radiat. Oncol., Biol., Phys. 81(2), S122 (2011).10.1016/j.ijrobp.2011.06.250

[10] E. J. Harris , N. R. Miller , J. C. Bamber , J. R. N. Symonds‐Tayler , and P. M. Evans , “Speckle tracking in a phantom and feature‐based tracking in liver in the presence of respiratory motion using 4D ultrasound,” Phys. Med. Biol. 55(12), 3363–3380 (2010).10.1088/0031‐9155/55/12/007 20505224 10.1088/0031-9155/55/12/007

[11] V. De Luca , M. Tschannen , G. Székely , and C. Tanner , “A learning‐based approach for fast and robust vessel tracking in long ultrasound sequences,” in Medical Image Computing and Computer‐Assisted Intervention–MICCAI 2013 (Springer Berlin Heidelberg, 2013), pp. 518–525. 10.1007/978-3-642-40811-3_65 24505706

[12] M. A. L. Bell , H. T. Sen , I. Iordachita , P. Kazanzides , and J. Wong , “ *In vivo* reproducibility of robotic probe placement for a novel ultrasound‐guided radiation therapy system,” J. Med. Imaging 1(2), 025001 (2014).10.1117/1.JMI.1.2.025001 10.1117/1.JMI.1.2.025001 PMC4479033 26158038

[13] M. von Siebenthal , G. Székely , A. J. Lomax , and P. C. Cattin , “Systematic errors in respiratory gating due to intrafraction deformations of the liver,” Med. Phys. 34(9), 3620–3629 (2007).10.1118/1.2767053 17926966 10.1118/1.2767053

[14] A. Gastounioti , S. Golemati , J. Stoitsis , and K. S. Nikita , “Comparison of Kalman‐filter‐based approaches for block matching in arterial wall motion analysis from B‐mode ultrasound,” Meas. Sci. Technol. 22(11), 114008 (2011).10.1088/0957‐0233/22/11/114008

[15] R. E. Kalman , “A new approach to linear filtering and prediction problems,” J. Basic Eng. 82(1), 35–45 (1960).10.1115/1.3662552

[16] R. Penoyer , “The alpha‐beta filter,” C. Users J. 11(7), 73–86 (1993).

[17] A. H. Hasan and A. N. Grachev , “Adaptive α‐β‐filter for target tracking using real time genetic algorithm,” J. Electr. Control Eng. (JECE) 3, 32–38 (2013).

[18] K. Saho , “Fundamental properties and optimal gains of a steady‐state velocity measured α‐β tracking filter,” Adv. Remote Sens. 3, 61–76 (2014).10.4236/ars.2014.32006

[19] http://clust.ethz.ch/data.html.

[20] T. R. Benedict and G. W. Bordner , “Synthesis of an optimal set of radar track‐while‐scan smoothing equations,” in IRE Transactions On Automatic Control (The Institute of Radio Engineers, New York, NY, 1962), pp. 27–32.

[21] G. C. Sharp , S. B. Jiang , S. Shimizu , and H. Shirato , “Prediction of respiratory tumour motion for real‐time image‐guided radiotherapy,” Phys. Med. Biol. 49(3), 425–440 (2004).10.1088/0031‐9155/49/3/006 15012011 10.1088/0031-9155/49/3/006

[22] S. J. Lee and Y. Motai , “Prediction and classification of respiratory motion,” in Studies in Computational Intelligence (Springer‐Verlag, Berlin, Heidelberg, 2014), p. 525.

[23] A. E. Lujan , E. W. Larsen , J. M. Balter , and R. K. Ten Haken , “A method for incorporating organ motion due to breathing into 3D dose calculations,” Med. Phys. 26(5), 715–720 (1999).10.1118/1.598577 10360531 10.1118/1.598577

[24] B. J. Geiman , L. N. Bohs , M. E. Anderson , S. M. Breit , and G. E. Trahey , “A novel interpolation strategy for estimating subsample speckle motion,” Phys. Med. Biol. 45(6), 1541–1552 (2000).10.1088/0031‐9155/45/6/310 10870709 10.1088/0031-9155/45/6/310

[25] I. Matthews , T. Ishikawa , and S. Baker , “The template update problem,” IEEE Trans. Pattern Anal. Mach. Intell. 26(6), 810–815 (2004).10.1109/TPAMI.2004.16 18579941 10.1109/TPAMI.2004.16

[26] M. M. Doyley , J. C. Bamber , T. Shiina , and M. O. Leach , “Reconstruction of elastic modulus distribution from envelope detected B‐mode data,” IEEE Ultrasonics Symposium (IEEE, 1996), p. 1611.

[27] T. Varghese and J. Ophir , “Characterization of elastographic noise using the envelope of echo signals,” Ultrasound Med. Biol. 24(4), 543–555 (1998).10.1016/S0301‐5629(98)00008‐8 9651964 10.1016/s0301-5629(98)00008-8

[28] Y. T. Oh , Y. Hwang , J. B. Kim , W. C. Bang , J. D. Kim , and C. Y. Kim , “Patient‐specific liver deformation modeling for tumor tracking,” Proc. SPIE 8671, 86711N (2013).10.1117/12.2007884

[29] C. Weon , W. H. Nam , D. Lee , J. Y. Lee , and J. B. Ra , “Position tracking of moving liver lesion based on real‐time registration between 2D ultrasound and 3D preoperative images,” Med. Phys. 42(1), 335–347 (2015).10.1118/1.4903945 25563273 10.1118/1.4903945

